# Correction: Ten Simple Rules for Taking Advantage of Git and GitHub

**DOI:** 10.1371/journal.pcbi.1007142

**Published:** 2019-06-14

**Authors:** The PLOS Computational Biology Staff

## Notice of republication

This article was republished on July 26, 2016, to correct errors in Tables 1 and 2 that were introduced during the typesetting process. The publisher apologizes for the errors. Please download this article again to view the correct version.

## Supporting information

S1 File Republished, corrected article. (PDF)
