# Supplementary material for: Ten Simple Rules for Taking Advantage of Git and GitHub
Source: PLoS Comput Biol. 2016 Jul 14;12(7):e1004947. doi: 10.1371/journal.pcbi.1004947 (PMC4945047; doi:10.1371/journal.pcbi.1004947)
Supplement: S1 File — (PDF) [file pcbi.1004947.s001.pdf]

Ten Simple Rules for taking advantage of git and GitHub: Supplementary File S1

Section 1: Git Large File Storage (LFS)

GitHub supports all types of files independently of their extension or content. If the file included in your repository is bigger than 50 MB, it should be committed using the Git LFS (Large File Storage) system. A minimal space quote is provided for personal and/or organization repositories without charge (1 GB at the moment of writing). If this quota is exceeded, you can still clone repositories containing large files, but you will only retrieve the “pointer” files. In order to use the git LFS service, users should download the git plugin from https://git-lfs.github.com/. Then, the following steps should be followed:

```
git lfs track "*.psd"
git add file.psd
git commit -m "Add design file"
git push origin master
```

Section 2: Testing Levels of the Source Code and Continuous integration

Software testing can be split in different categories or levels of complexity, ranging from unit tests to system testing. We here explain in a bit more detail unit and integration tests, which in our opinion represent the starting point to provide a functional software.

Unit tests can be used for individual units of source code, or sets of one or more modules together with associated control data, usage procedures, and operating procedures. Ultimately, this helps to identify flaws and mistakes in the algorithms and/or the logic. These types of tests are usually written by developers as they work in the code (following a white-box style), to ensure that the specific function is working as expected. Furthermore, one function might have multiple tests, to consider different use cases or functionalities in the code. Unit testing alone cannot verify the functionality of a piece of software, but it is rather used to ensure that the building blocks of the software can work independently from each other.

```
public class TestPTM {

    // can it add the positive numbers 1 and 1?
    public void testPTMIdentifier() {
        PTM ptm = new PSIModPTM();
        assert(ptm.parent.add(1, 1) == 2);
    }
}
```

Unit testing can detect problems early in the development cycle. These can include both bugs in the programmer’s implementation, and flaws or missing parts of the specification for the unit. The process of writing a thorough set of tests forces the programmer to think about inputs, outputs, and error conditions, thus defining more crisply the desired behavior of the unit. Some people argue that code which is impossible or difficult to test is poorly written. Therefore unit testing can force developers to structure functions and objects in better ways. The unit tests can also be more complex to explore the *logic* of the code by testing the expected results of a specific algorithm or mathematical model.

```java
@Test
public void TestGetPTms() {
     List<PTM> ptms = modReader.getPTMListByPatternDescription("Phospho");
     assertTrue("Number of PTMs with Term 'Phospho' in name:", ptms.size() == 106);
}
```

However, when your software is run, all those units and modules have to work together, and the software as a whole is more complex than the sum of its independently-tested parts. Proving that components X and Y can both work independently does not prove that they are compatible with one another or that are configured correctly. Problems during this interaction may have no relation to the behaviour that an end user would experience and report. If you can automate this sort of 'component interaction', by performing tests in order to detect breakages when they happen in the future, this is called integration testing. Typically it makes use of different techniques and technologies than unit testing.

Integration testing represents the phase in which individual software modules are combined and tested as a group. It occurs after unit testing and before validation testing. Integration testing takes as input the modules that have been unit tested before, groups them in larger aggregates, then applies to those aggregates tests defined in an integration test plan, and delivers the integrated system ready for system testing.

Using Travis-CI users can perform integration testing of their software and the corresponding dependencies. See the following example from the pIR R package (available at https://github.com/ypriverol/pIR/):

```yaml
sudo: required
language: c

before_install:
  - curl -OL http://raw.github.com/craigcitro/r-travis/master/scripts/travis-tool.sh
  - chmod 755 ./travis-tool.sh
  - ./travis-tool.sh bootstrap

install:
  - ./travis-tool.sh install_bioc BiocInstaller
  - ./travis-tool.sh install_deps
  - ./travis-tool.sh r_install knitr

script: ./travis-tool.sh run_tests

on_failure:
  - ./travis-tool.sh dump_logs

notifications:
  email:
    on_success: ypriverol@gmail.com
    on_failure: ypriverol@gmail.com

after_failure:
  - ./travis-tool.sh dump_logs
```

The provided example installed all the dependencies of the library (BiocInstaller) and run all the corresponding tests in the R package software. More documentation about the Travis-CI integration can be found at https://travis-ci.org/ .

Section 3: Source code documentation

From our point of view, documentation of the source code should mainly introduce details about the methods, the rationale behind the algorithms and their possible flaws. The documentation should be self-explanatory and certainly should not include any “noise”. For example, the following example represents in our opinion a bad practice:

```
for( int i = 0 ; i < list.size(); i++)  // "loop across all the elements of the list.
```

The previous example represents the same information in the source code and in the comment. Some developers argue that if you have a 1 to 1 or even a 5 to 1 ratio between the lines of code (LOC) and comment lines, there are probably too many comment lines. In fact, the need for excessive comments is a good indicator that your code may need refactoring.

Some developers use the comment section to explain decisions in the code and also to indicate who actually took the corresponding decision:

```
// Revisions: Sue (2/19/2014) - Lengthened monkey's arms
//            Bob (2/20/2015) - Solved drooling issue

    void pityTheFoo() {
```

In contrast to the previous example, the following code is, in our opinion, an example of good practice:

```
/**
     * Returns <tt>true</tt> if this map maps one or more keys to the
     * specified value.  More formally, returns <tt>true</tt> if and only if
     * this map contains at least one mapping to a value <tt>v</tt> such that
     * <tt>(value==null ? v==null : value.equals(v))</tt>.  This operation
     * will probably require time linear in the map size for most
     * implementations of the <tt>Map</tt> interface.
     *
     * @param value value whose presence in this map is to be tested
     * @return <tt>true</tt> if this map maps one or more keys to the
     *         specified value
     * @throws ClassCastException if the value is of an inappropriate type for
     *         this map
     * (<a href="Collection.html#optional-restrictions">optional</a>)
     * @throws NullPointerException if the specified value is null and this
     *         map does not permit null values
     * (<a href="Collection.html#optional-restrictions">optional</a>)
     */
    boolean containsValue(Object value);
```

The example, coming from the Java Application Programming Interface (API), provides information about the method, what it should return and the possible exceptions related with the behaviour of the *Map* collection. In Table 1 in the main manuscript we provide some useful links to documentation about best practices and styles. If the source code is well documented, different tools have been integrated in GitHub that can be used to generate the final version of the documentation. Some examples are Sphinx (http://www.sphinx-doc.org/) and “Read the Docs” (https://readthedocs.org/).
However, we strongly suggest that the documentation of the source code should be actively maintained as the

source code itself. In fact, when a piece of code is changed the corresponding documentation and comments should be inmediately reviewed.

Finally, below, we provide some useful documentation about source code comments and documentation:

- 13 Tips to Comment Your Code. http://www.devtopics.com/13-tips-to-comment-your-code/.
- How to Write Doc Comments for the Javadoc Tool. http://www.oracle.com/technetwork/articles/java/index-137868.html.
- Do Code and Comments Co-Evolve? On the Relation between Source Code and Comment Changes. http://dl.acm.org/citation.cfm?id=1339530.
- CodeAsDocumentation. http://martinfowler.com/bliki/CodeAsDocumentation.html.
